# The Clinical Role of CT-Based Morphologic Description in Severely Calcified Coronary Arteries Ectasia Encountering Acute Coronary Syndrome

**DOI:** 10.1155/2012/267496

**Published:** 2012-02-26

**Authors:** Yen-Yu Liu, Jui-Peng Tsai, Chung-Lieh Hung, Jiun-Yi Li, Jen-Yuan Kuo

**Affiliations:** ^1^Division of Cardiology, Department of Internal Medicine, Mackay Memorial Hospital, Taipei 25160, Taiwan; ^2^Mackay Medicine, Nursing and Management College, Taipei 112, Taiwan; ^3^Division of Cardiovascular, Department of Surgery, Mackay Memorial Hospital, Taipei 25160, Taiwan

## Abstract

Diffuse coronary arteries ectasia combined with calcification is seldom reported. Acute coronary syndrome, a potentially life-threatening disease, accompanied with coronary ectasia and diffuse calcification, made percutaneous coronary intervention difficult and risky owing to increasing complications rate. Dual-source computed tomography and three-dimensional volume rendering images help cardiovascular surgeon easier to localize the ideal site and facilitate the procedure.

## 1. Introduction

Coronary artery ectasia (CAE) is anatomical abnormality in coronary artery tree with localized or diffuse dilatation, which has been found in 0.3–5% of coronary angiography. Vascular calcification is an important issue in dialysis patients and had been documented to be associated with cardiovascular diseases. Atherosclerosis is the leading cause of coronary ectasia and is associated with intimal calcification. Not only vascular ectasia but also calcification complicated treatment, especially in acute coronary syndrome (ACS). We reported a case of coexisting CAE and diffuse calcification encountering ACS. We performed coronary angiography for diagnosis and vascular computed tomography with three-dimensional reconstruction image to assist surgical revascularization.

## 2. Case Report

In April 2011, a 41-year-old man who was a heavy smoker visited our emergency department presenting with acute onset of compressive chest pain, jaw soreness, and cold sweats while watching television. His medical history included end-stage renal disease on continuous ambulatory peritoneal dialysis for the past 5 years and secondary hyperparathyroidism status after parathyroidectomy and autoreimplantation in 2009.

**Figure 1:**
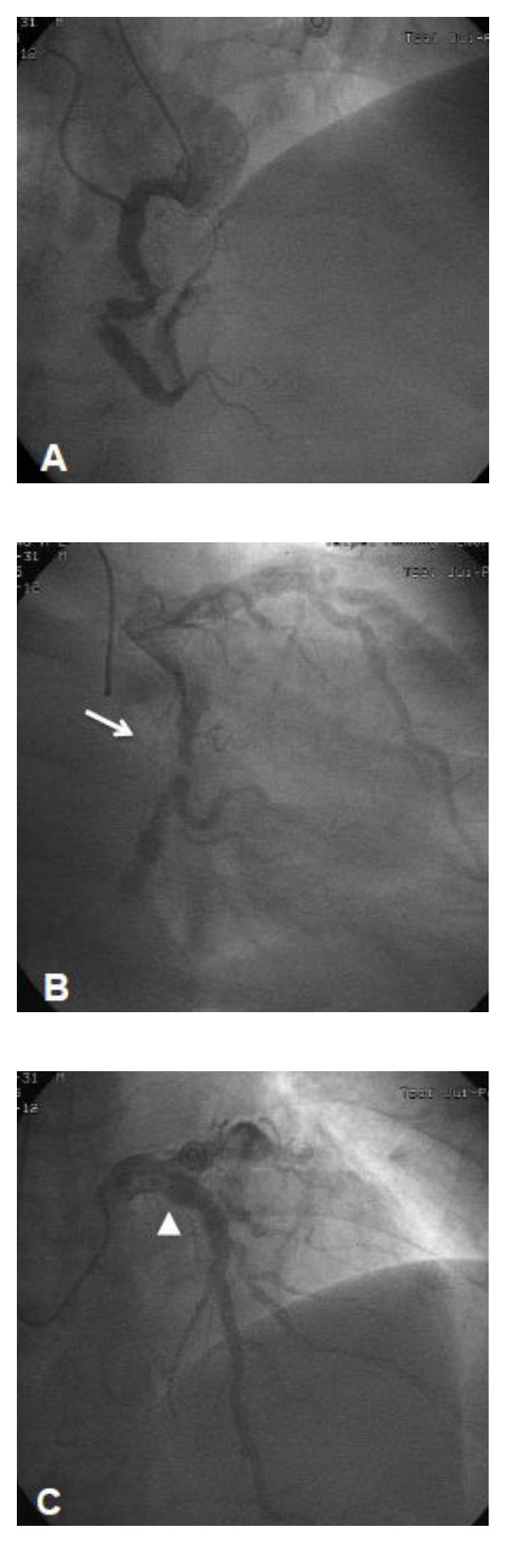
Coronary angiography revealing ectasia in the (A) right coronary artery, (B) left circumflex artery (arrow), and (C) left descending artery (arrow head).

**Figure 2:**
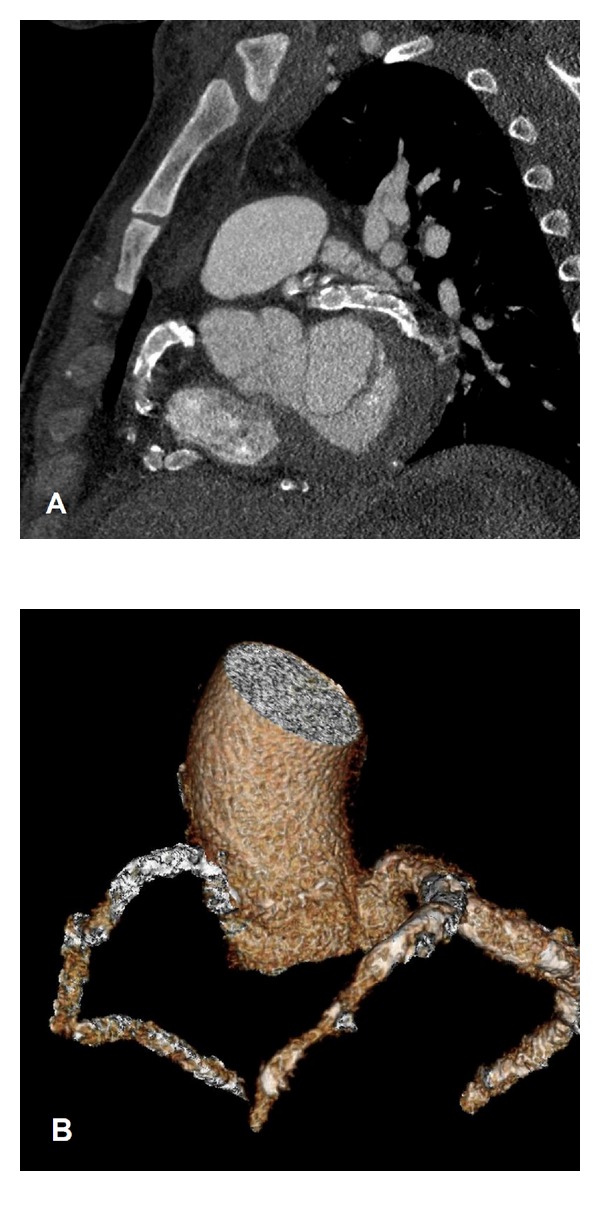
Dual source computed tomography showing (A) diffuse calcification in RCA and LCx and (B) 3-dimensional volume rendering image demonstrating coronary arteries ectasia.

**Figure 3:**
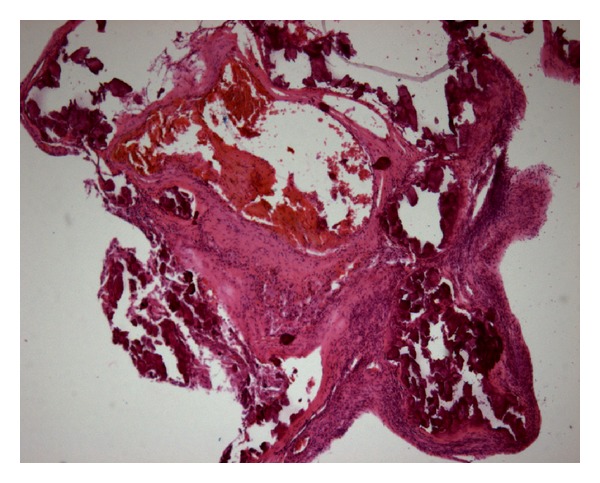
Coronary endarterectomy showed atherosclerotic plaques and calcification.

On examination, blood pressure was 120/73 mmHg, heart rate was 88 beats per minute, and respiratory rate was 20 per minute. The chest X-ray showed slight cardiomegaly and pulmonary congestion. His initial surface 12-lead electrocardiogram (ECG) at admission showed normal sinus rhythm, left ventricular hypertrophy by voltage criteria, and ST segment depression at lateral wall territory. Levels of Troponin I and creatinine kinase-MB were 2.32 ng/mL (normal value, <0.5 ng/mL) and 20.5 ng/mL (normal value, <5.4 ng/mL), respectively. Transthoracic echocardiography disclosed with ejection fraction of 59% and regional wall motion abnormality in anterior and basoinferior segments of left ventricle. Under the tentative diagnosis of acute coronary syndrome (ACS), low-molecular weight heparin, aspirin, and clopidogrel were administered subsequently. We put a 6 French sheath by left radial artery and administered 5 French Judkin right and Judkin left coronary catheters to perform emergency coronary angiography (CAG), which demonstrated diffuse ectasia and heavy calcification of all three coronary arteries with nearly total occlusion of the mid right coronary artery (RCA) (Figure 1(A)) and the mid left circumflex coronary artery (LCX) (Figure 1(B)) accompanied by a 90% luminal narrowing at the first diagonal branch and an 80% stenosis of the second diagonal branch of left anterior descending coronary artery (Figure 1(C)). All coronary arteries had grade 2 flow by thrombolysis in myocardial infarction definition. The RCA and LCX were tortuous. Due to the difficulty in applying intravascular ultrasound while approaching coronary arteries, coronary artery bypass grafting (CABG) was suggested instead of percutaneous coronary intervention. Dual-Source computed tomography (DSCT) with ECG synchronization of the heart (Figure 2(A)) was done. Interestingly, we observed that diffuse calcifications mainly focus on the coronary arteries sparing ascending aorta, which was further confirmed by subsequent cardiovascular surgical exploration. Three-dimensional (3D) volume rendering images were also obtained in order to reconstruct a more comprehensive view in identifying the proper anatomy for possible vessel anastomosis during CABG surgery (Figure 2(B)). Cardiopulmonary bypass with cardioplegic cardiac arrest was applied. The first segmented saphenous vein graft (SVG) was implanted sequentially from aorta to obtuse marginal branch, posterior left ventricular branch, and posterior descending artery. The second segmented SVG was anastomosed from the side of first SVG to diagonal branch and LAD. The patient got extubated 24 hours after the operation and remained neurologically intact. The postoperative recovery course was uneventful, and he was discharged from hospital in 10 days being symptom-free. The pathologic study reported atherosclerotic plaques and calcification of the coronary arteries (Figure 3).

## 3. Discussion

The prevalence of coronary artery disease (CAD) among patients receiving hemodialysis or peritoneal dialysis is approximately 40% and 75%, respectively [1]. In dialysis patients, cardiovascular disorders are the major cause of death and account for 43% of all-cause mortality in this population. The incidence of sudden cardiac death in ESRD patient was nearly 7% annually owing to obstructive coronary artery diseases [2], which may be contributed to accelerated vascular calcification and CAD [3]. Several factors, including abnormalities in serum phosphate, serum calcium, calcium phosphate product, C-reactive protein, low fetuin-A level, and malnutrition [4], had been documented to be associated with vascular calcification. In recent study, serum fetuin-A level was associated with cardiovascular mortality in uremia patients [5]. Moreover, C-reactive protein plays a role in initiation and progression of accelerated atherosclerosis in dialysis patients [4].

Furthermore, atherosclerosis remained the leading cause of CAE, followed by Kawasaki's disease and infectious septic emboli, which is found in 0.3–5% of patients undergoing coronary angiography [6]. Passive deposition of calcium and phosphate due to abnormal bone tissue metabolism and impaired renal excretion in CKD may lead to differentiation of vascular smooth muscle cells into osteoblastic cells with subsequent ossification of the arterial wall. The calcification in the tunica intima, a marker of vascular atherosclerosis, further accelerates plaque growth along with enzymatic degradation of the extracellular matrix of the media with excessively vascular remodeling leading to coronary artery ectasia development [7]. According to coronary artery surgery study (CASS) registry, CAE was defined as a diameter ≥1.5 time of the reference vessel size [8]. Markis et al. [9] classified CAE based on ectatic involvement, in decreasing order of severity and frequency, as follows: (1) diffuse ectasia involving two or three vessels (type I), (2) diffuse ectasia involving one vessel and localized ectasia in another (type II), (3) diffuse ectasia in one vessel only (type III), and (4) localized ectasia in one vessel only (type IV). Baman et al. [10] declared clinicians should regard aneurysmal coronary disease as a predictor of mortality and adjust cardiovascular risks aggressively. Isolated ectatic vessel without stenosis could be benefited from urokinase infusion in acute thrombotic occlusion [11], while aspirin and warfarin were administered in CAE coexisting with coronary stenosis [8, 12]. Surgical revascularization is also recommended in such situation. With the help of intravascular ultrasound, percutaneous intervention could be performed safely with adequate vessel assessment [13].

Although CAE has been reviewed before [5], to our best knowledge, its combination with diffuse calcification has seldom been reported in previous literature. Here, we report a case of diffuse calcification and ectasia with several sites of critical stenosis in the coronary arteries in an ESRD patient manifesting as ACS. Our patient had ectasia in three coronary arteries as type I in CASS classification. Diffuse calcification and tortuous vessels made percutaneous coronary intervention difficult and risky. Preoperative CT could fully explore the geometric characteristics of the coronary arteries. CT-based reconstruction of 3-dimensional volume rendering imaging could further facilitate surgical approach in localizing the optimal sites for CABG.

In conclusion, this is a rare case of coronary arteries ectasia combined with diffuse calcification presenting with ACS in an ESRD patient. In particular, the specialized images not only illustrated the spatial characteristics of the severely calcified coronary arteries, but also helped in planning the ideal sites for bypass grafting anastomosis preoperatively.
